# Onset of Immune Senescence Defined by Unbiased Pyrosequencing of Human Immunoglobulin mRNA Repertoires

**DOI:** 10.1371/journal.pone.0049774

**Published:** 2012-11-30

**Authors:** Florian Rubelt, Volker Sievert, Florian Knaust, Christian Diener, Theam Soon Lim, Karl Skriner, Edda Klipp, Richard Reinhardt, Hans Lehrach, Zoltán Konthur

**Affiliations:** 1 Max Planck Institute for Molecular Genetics, Berlin, Germany; 2 Faculty of Biology, Chemistry, and Pharmacy, Freie Universität Berlin, Berlin, Germany; 3 Theoretische Biophysik, Humboldt-Universität zu Berlin, Berlin, Germany; 4 Institute for Research in Molecular Medicine, Universiti Sains Malaysia, Penang, Malaysia; 5 Department of Rheumatology and Clinical Immunology, Charité – Universitätsmedizin Berlin, Berlin, Germany; 6 Max Planck Genome Centre Cologne, Max Planck Institute for Plant Breeding Research, Cologne, Germany; University of Medicine and Dentistry of New Jersey - New Jersey Medical School, United States of America

## Abstract

The immune system protects us from foreign substances or pathogens by generating specific antibodies. The variety of immunoglobulin (Ig) paratopes for antigen recognition is a result of the V(D)J rearrangement mechanism, while a fast and efficient immune response is mediated by specific immunoglobulin isotypes obtained through class switch recombination (CSR). To get a better understanding on how antibody-based immune protection works and how it changes with age, the interdependency between these two parameters need to be addressed. Here, we have performed an in depth analysis of antibody repertoires of 14 healthy donors representing different gender and age groups. For this task, we developed a unique pyrosequencing approach, which is able to monitor the expression levels of all immunoglobulin V(D)J recombinations of all isotypes including subtypes in an unbiased and quantitative manner. Our results show that donors have individual immunoglobulin repertoires and cannot be clustered according to V(D)J recombination patterns, neither by age nor gender. However, after incorporating isotype-specific analysis and considering CSR information into hierarchical clustering the situation changes. For the first time the donors cluster according to age and separate into young adults and elderly donors (>50). As a direct consequence, this clustering defines the onset of immune senescence at the age of fifty and beyond. The observed age-dependent reduction of CSR ability proposes a feasible explanation why reduced efficacy of vaccination is seen in the elderly and implies that novel vaccine strategies for the elderly should include the “Golden Agers”.

## Introduction

**Figure 1:**
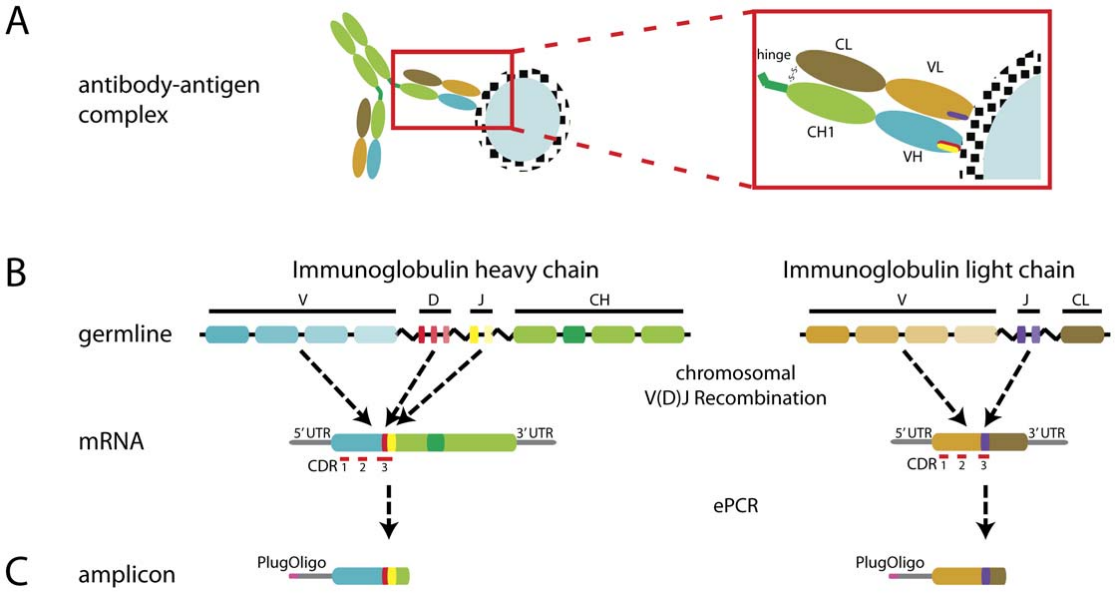
Schematic illustration of immunoglobulin G in complex with antigen and mechanism of V(D)J recombination, as well as amplification strategy for 454-sequencing. (**A**) Immunoglobulin-antigen interaction. Immunolgobulins recognize antigens via paratopes primarily defined by complementarity determining regions (CDRs). VH: Variable heavy; VL: Variable light; CH1 Constant heavy 1; CL: Constant light. (**B**) Representation of the genomic rearrangements occurring during V(D)J recombination. Chromosomal V(D)J rearrangement in the B-cell connect one Variable-gene (V) with one Diversity (D; only HC) and one Joining-gene (J) out of a pool of different V(D)J genes to enable a high diversity of binding affinities within the antibody repertoire. CDR 1 and 2 are defined by the V-genes, while CDR 3 is generated through V(D)J recombination. Constant domains (CH and CL) specify the induced immune reaction. (**C**) Amplicon represent dsDNA fragments generated for pyrosequencing by emulsion PCR (ePCR). PlugOligo: V-gene independent 5′ end adapter for amplification.

The humoral immune system creates a vast diversity of immunoglobulins (Ig) via rearrangements of variable- (V), diversity- (D; only in heavy chain) and Joining- (J) gene segments [1] to generate a pool of antibodies being able to bind to foreign substances or pathogens (Figure 1). Once an antigen is entering the body, an initial IgM-response is affinity-matured by somatic hypermutation and is finally transferred into an immune response mediated by specific immunoglobulin isotypes obtained through class switch recombination (CSR) [2]. Hence, to get a better understanding of antibody-based immune protection it is not enough to assess V(D)J recombination, but the effector function of an antibody encoded in the isotype is of equal importance. All antibody classes have different functions and the switch from IgM/IgD to a different isotype is a controlled and complex process [3].

In depth analysis of antibody repertoires of healthy donors representing different age groups has not been performed yet, although it is of major interest for the understanding of reduced vaccination efficacy in elderly populations [4], [5]. Recent findings suggest that the dramatically reduced vaccination efficacy in elderly populations is not because of a lack of specific antibodies due to reduction of V(D)J recombination, but rather a problem in antibody titre and lacking specificity in the right immunoglobulin class to elicit an adequate response [6].

In our study we set out to monitor for the first time V(D)J recombination patterns interrelated with Ig-isotype information on an mRNA level using Next Generation Sequencing (NGS) in an unbiased and quantitative manner. NGS has revolutionized the research on antibody repertoires by providing a before unreached amount of antibody sequences for analysis. NGS was first employed for the analysis of Ig heavy chain repertoires in the Zebrafish model [7], [8]. Since then, multiple insights into the nature of antibody diversity has been provided in an unrivalled depth focusing on specific questions, however, primarily investigating only into fractions of the Ig-repertoire [9]–[18]. Standard amplification of Ig-repertoires from mRNA use many different V-gene specific primers in parallel reactions to ensure completeness [14], [19], [20]. To diminish possible primer-dependent bias [21], we developed a novel amplification strategy independent of V-gene specific 5′ primers. Further, our novel avenue of analysis is based not only on information on V(D)J recombination but also on CSR profiles of individual donors by incorporating isotype-specific analysis of the antibody sequences. As a direct consequence, donors clustered hierarchically according to age. For the first time we could observe changes in immunoglobulin isotype repertoires to be age-dependent indicating reduction of class switch recombination ability already occurring at a much earlier time point than expected.

## Results and Discussion

### Unbiased amplification and sequencing of human Ig-repertoires

We have developed a novel amplification strategy for heavy and light chain (HC and LC) repertoires starting from total RNA of peripheral blood cells. We used a single V-gene independent 5′ end adapter (PlugOligo) during reverse transcription in combination with five HC and two LC PCR primers derived from conserved CH1/CL regions (Figure 1). CH1-specific primers were chosen in such a way that the obtained sequences could be subsequently subdivided into five isotypes with nine subtypes (IgA1, -A2, -D, -E, -G1, -G2, -G3, -G4, -M). Since PCR-based amplification processes can skew the Ig-repertoire, we developed a “single-pot” emulsion-based method for HC and LC amplification to ensure unbiased amplification and maintenance of diversity [21]. DNA sequencing of Ig-repertoires from 14 healthy Caucasians of different age and gender was performed using a Roche Genome Sequencer FLX/454 system [22]. In total 3,566,089 reads were obtained. The raw sequences were analyzed according to three criteria (i) over 380 bp length, (ii) unique assignment to Ig-class and (iii) unambiguous assignment of V(D)J rearrangements to V-, D- and J-genes. For assignment of rearrangements we applied the IMGT/High V-Quest tool [23]–[25] and for class assignment we developed and employed a signature-based method independent of the CH1-specific primers used for amplification. Classification of sequences was performed on a gene rather than allele resolution using regular expression pattern matching of the IMGT/High V-Quest output supplemented with a heuristic approach to handle genes, which could not be identified unambiguously. A relational data model was developed for structural storage retrieval of both raw sequence data and analysis results based on the PostgreSQL RDBMS.

In total, we obtained 1,357,978 (38.08%) sequences with a complete and unambiguous set of V(D)J gene assignments of high quality (1,046,521 HC and 295,555 LC). Our method allows the unbiased analysis of V-gene usage in antibody repertoires and its power is demonstrated by obtaining sequences of several V-genes, e.g. IGHV3-13 and IGHV4-61, which have been missed in previous deep sequencing studies where V-gene specific primers were applied [12]. The analysis of light chain repertoires revealed that the most frequently used kappa and lambda light chains over all donors are IGKV1-39 and IGKV4-1 (14% and 13%) and IGLV2-14 and IGLV1-40 of (22% and 8%), respectively.

### Analysis of V(D)J recombination in healthy donors of different age and gender

**Figure 2:**
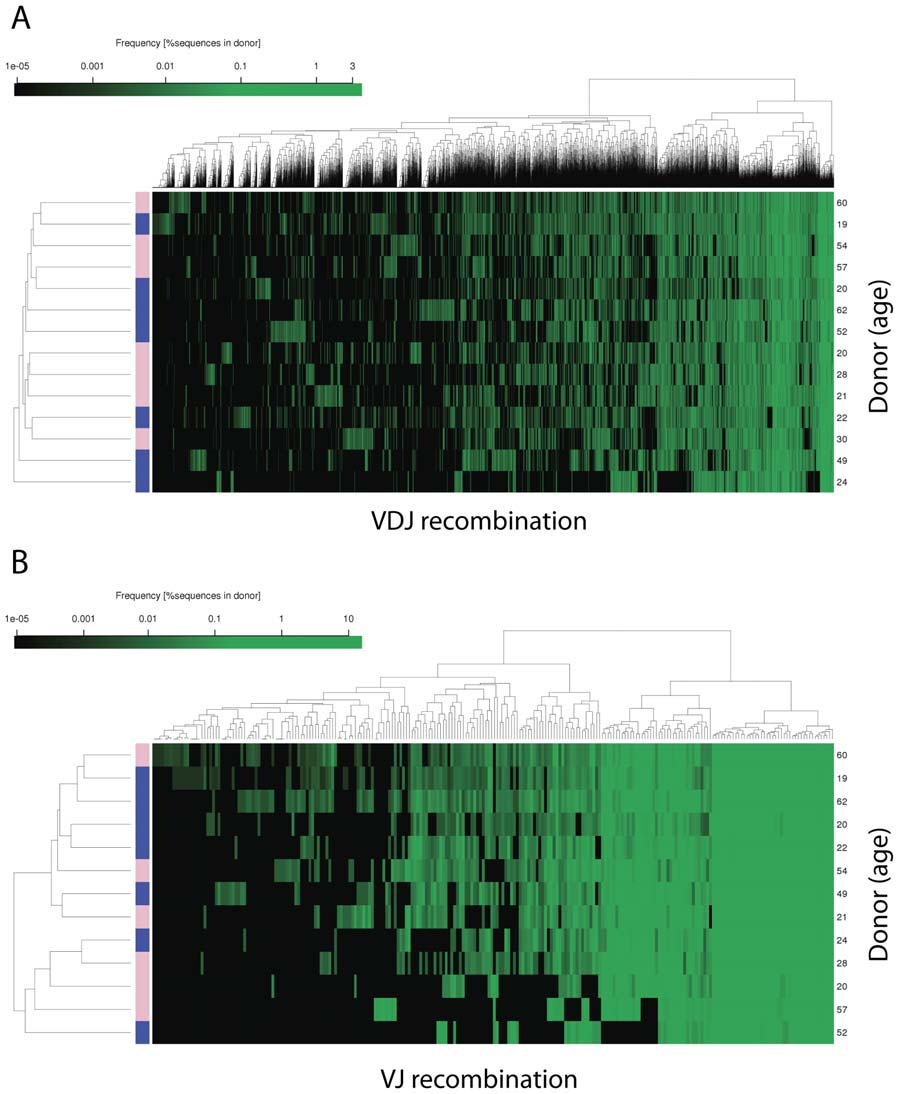
Hierarchical clustering of V(D)J recombination pattern distributions. (**A**) Clustering of HC VDJ rearrangements. (**B**) Clustering of LC VJ rearrangements. Heatmaps show relative frequency of V(D)J recombination patterns (columns) versus donors (rows). Blue and pink colors represent male and female, respectively. The age of the donor is recorded on the right. Individual V(D)J counts were normalized by total number of sequences for each donor. Normalized frequencies were log-transformed [*F* as ln(*F*+1e-6)] and intensity was visualized from black to lime. Row and column dendograms use euclidean distance.

**Figure 3:**
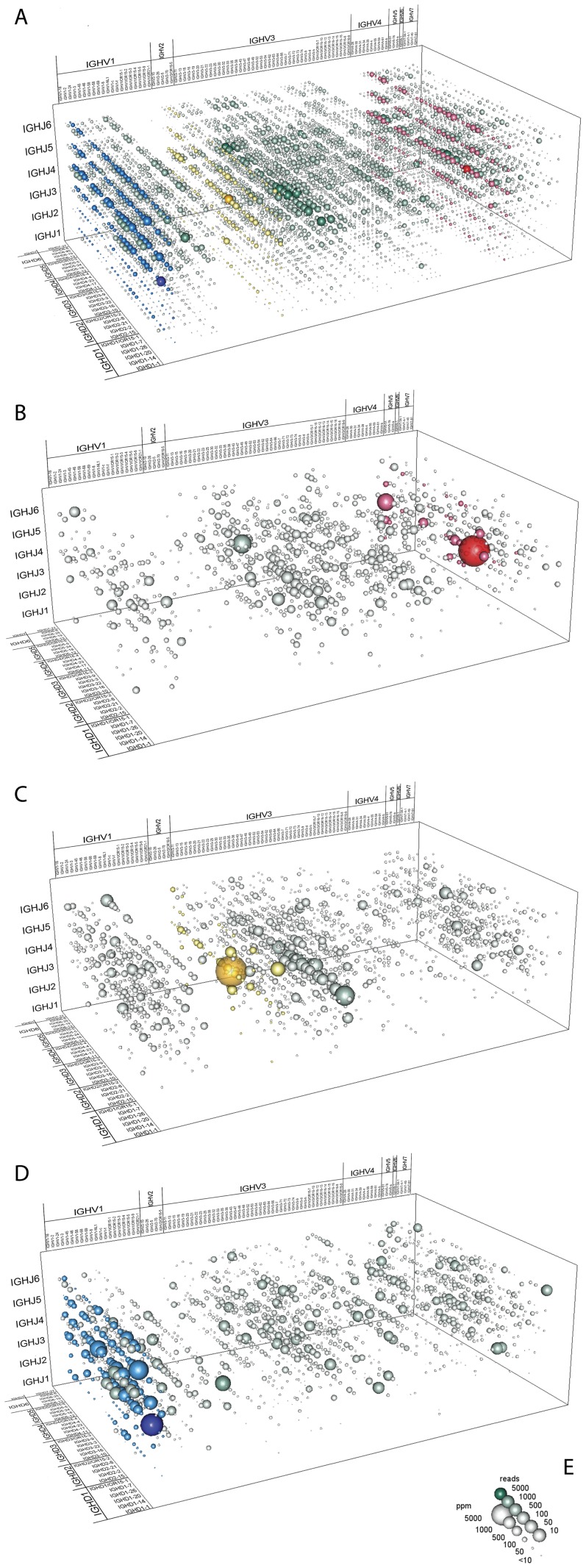
In detail analysis of VDJ rearrangements. (**A**) Overall distribution of VDJ rearrangements in 14 donors. (**B**) IgA2-specific VDJ rearrangements in donor I200091-032. (**C**) IgA1-specific VDJ rearrangements in donor I200091-030. (**D**) IgG1-specific VDJ rearrangements in donor I200091-021. (**E**) Gauge; sphere volumes refer to their respective numerical proportion. Less than 10 ppm are represented by a fixed size sphere. Green color shading indicates number of reads constituting respective recombination. Other colors highlight selected V-genes; blue: IGHV1-2, yellow: IGHV2-5, red: IGHV4-34.

The description of V(D)J recombination frequencies to reflect complete antibody repertoires is inherently complex. Looking only at the combination of individual V-, D- and J-gene segments independent of the resulting antibody sequence, we already found 6685 different VDJ recombination patterns for HC and 240 different VJ recombination patterns in LC. We performed hierarchical clustering of the 14 donors based on the overall distribution and relative frequency of these V(D)J recombination in HC and LC and found neither age nor gender-specific grouping (Figure 2). Next, we included isotype information and repeated the clustering (Text S1). Again, no grouping was observed (Figure S1). At the same time, however, we observed an overall tendency in the usage of certain VDJ recombinations within the 14 individuals of the cohort (Figure 3A) and analyzed the most frequent rearrangements; those present 100-times over the median expression of all VDJ recombination (>1.07%; Figure S2). These VDJ recombination patterns were predominantly found in only single isotypes suggesting to have originated from oligoclonal expansion of B-cells and, hence, reflecting natural diversification of specific immune responses. The three most frequent VDJ-rearrangements were analyzed in greater detail and strikingly, each of them could almost exclusively be assigned to one major Ig-isotype. Donor I200091-032 showed a distinct recombination pattern (VH4-34/D2-12/J4; 3.4%), of which 96% is expressed as IgA2 (Figure 3B). A detailed analysis of the complementarity determining regions (CDRs) on the amino acid level further revealed that 65% of this VDJ rearrangement can be attributed to two sequences (54% and 11%, respectively) that differ in a single amino acid. This could be either a result of clonal expansion or of a kind of converging maturation in response to an antigen. Donor I200091-030 has an elevated VH2-5/D3-22/J4 recombination pattern (3.8%) with a frequency of 95% in isotype A1 (Figure 3C). Detailed examination of CDR-composition clearly suggests a polyclonal response since an even distribution of seven different amino acid sequences contribute to 60% of this isotype recombination. In the third example (donor I200091-21, Figure 3D), the most frequent recombination VH1-2/D1-26/J3 (3.3%) was observed within IgG1 (89%). At the same time we noticed in this donor a broad usage of VH1-2 (21.6%) with different J and D segments over all V-genes of which the majority was seen in IgG1 (17.6% of all). Altogether, IgG1 expression was 8 times over the median of all donors in this case indicating a polyclonal or multi-antigenic immune response since no distinct amino acid pattern was observed on the CDR-level. Although none of the donors analyzed were vaccinated recently and all were asymptomatic and without any recent or long-term medical pre-history according to voluntary disclosure, the determination of the Ig-repertoires by NGS already implies that our method will be suitable to monitor V(D)J recombination patterns in response to specific antigens/vaccines or during the course of certain diseases, as suggested earlier [26].

### Ig-isotype frequencies show age-dependency

**Figure 4:**
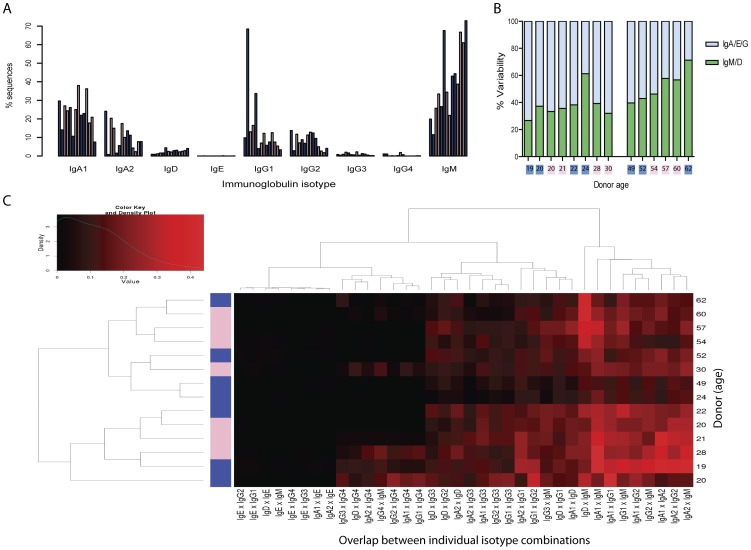
Analyses based on Ig-isotype distributions. (**A**) Relative frequency of isotype-specific sequences within donors sorted left to right according age. (**B**) Relative variability of isotypes on the basis of initial (IgM/D) and specific response (IgA/E/G). Variability: percentage of VDJs covered by a distinct isotype in each donor. Variability *V_AD_* for antibody type *A* and donor *D* was calculated from the number of occurring VDJs *n_AD_* and the total number of occurring VDJs in the donor *D n_D_* as *n_AD_/n_D_*. (**C**) Clustering of donors (rows) according to coincident appearance of most frequent VDJ rearrangements in their isotypes (column) with age and gender. The hundred most frequently occurring VDJ rearrangements (or less if there were less than hundred) for each donor and isotype were selected. For each donor the overlap between each pair of isotypes was quantified (visualized from black to red) using the formula *n_both_/*max(n_A_, n_B_), where *n_both_* is the number of VDJs present in both sets of VDJs and n_A_ and n_B_ represent the sizes of the sets. Blue and pink colors represent male and female, respectively.

Our method results in a quantitative overview of all isotypes and their relative abundance, offering a detailed picture of the immunoglobulin repertoire. At first we calculated for each donor the relative amount of obtained sequences per isotype over the total number of sequences. Most reads belonged to IgM (median 35.7%), whereas IgE- or IgG4-specific reads were rarely obtained. Looking at the cohort as a whole, a correlation between IgM (pval = 0.005) and IgD (pval = 0.029) expression levels and age of the donors could readily be observed (Figure 4A; Table S1). After dividing the donors into young adults (19–30 years) and elderly (49–62 years) we found additionally a significant increase in the IgM (pval = 0.029) and a concomitant decrease in IgG2 (pval = 0.027) levels as well as an uneven reduction in the other isotypes among the elderly (except IgD and IgG4). At the same time, no correlation between isotype distribution and age was observed in the group of young adults (Tables S2 and S3). We noticed that the antibody repertoire of the 24 years old male resembles more that of elderly with a higher frequency of IgM and IgD than the other young adults.

### Differences in CSR-ability are observed between young and elderly

We calculated the number of unique VDJ recombinations per isotype in proportion to all isotypes for each donor (Figure S3). No age dependency in the young adults was seen and the relative number of unique VDJ recombinations for each isotype is comparable within this group. In the group of elderly, however, age dependency was clearly observed with a gradual increase in relative numbers of VDJ recombination in IgM (pval = 0.006) and IgD (pval = 0.003) and a significant decrease in IgG2 (pval = 0.011). This is in good agreement with the above finding purely based on isotype analysis and clearly relates to the biological function; i.e. dividing an immune reaction into initial response (IgM/D) and specific response (IgA/E/G) after class switch recombination (Figure 4B). Naïve B-cells initially express IgM or IgD through alternative mRNA splicing and only after stimulation with antigens will the cells undergo CSR of the antigen receptor resulting in IgA/E/G expressing B-cells through a process of DNA rearrangement driven by enzymatic processes [27]. CSR marks the onset of a specific response and results in changes in immunoglobulin effector function while the specificity of the immunoglobulin to the antigen, and hence variable domain usage, remains largely unaffected. We observed in the elderly a strong correlation between age and reduction in CSR ability (correlation 0.95; pval = 0.004) and no correlation in the young adults (pval = 0.663) (Tables S4, S5, S6).

### Hierarchical clustering segregates young and elderly

Finally, we compared the VDJ recombination patterns within the donors with regards to CSR by analyzing the overlap between VDJ recombination in the different isotypes. The results of the top 100 VDJ rearrangements between each isotype of a donor were applied to cluster the donors hierarchically. The mean of the overlap significantly differed in the young and elderly groups (p-value = 0.0112; Welch two sample t-test) and a reduction in the elderly of 35.52% (young: 0.1185; elderly: 0.0811) was observed, clearly segregating the two age groups. The heat map in Figure 4C shows that donors clustered according to age but not gender. Below the age of fifty, the donors clustered in pairs age-independently, while above fifty the donors with similar age cluster pairwise suggesting correlation according to reduced CSR ability. Clustering on the basis of single isotypes or group (e.g. IgGs) only, revealed a tendency for age correlation (Figures S4, S5, S6, S7). We conclude that only monitoring the complete repertoire, now possible for the first time, can reveal donor-specific implications of impairment of CSR and shed light into the complexity of immune senescence in the elderly.

### Analysis of changes in the VDJ rearrangement pattern distribution by entropy

Additionally, entropy was used as a measure of dispersion within the distribution of VDJ recombination for individual donors. In order to minimize the influence of sample size (number of available sequences) and the high number of possible recombinations in regimes of small sample sizes entropy was calculated according to the method of Chao and Shen [28], which takes unobserved species into account and performs mostly independent of sample sizes. Because entropy quantifies the maximum information a distribution can comprise, it can be used as another measure for the diversity of the antibody repertoire. Calculations were carried out over all donors as well as over the age groups separately (Tables S7, S8, S9, respectively). No correlation or significant values were obtained for the young adults. Consistent with the results for the variability, entropy is increasing with age for the IgM and IgD isotypes (IgM, pval = 0.034; IgD, pval = 0.005) and there is a decline in entropy within the IgG2 isotype in the elderly (pval = 0.009). Thus, the diversity of the immune response, particularly in the elderly, is retained within the IgM/D isotype and not adequately transferred to the IgG isotypes, which remain significantly less diverse (smaller entropy). This can be attributed to reduced CSR.

### The age of fifty and beyond marks the onset of immune senescence

Age has a strong influence on the repertoire of the isotypes due to onset of reduction of CSR from IgM/IgD to the more specific and effective isotypes IgA and IgG, which are important not only for rapid and efficient immune response to infectious agents [29] but also for the induction of immune protection upon vaccination. Decreased production of vaccine-specific antibodies rather than VDJ recombination efficiency or avidity is thought to be responsible for overall decrease of vaccine-efficacy [6], presumably due to impairment of CD4^+^ helper cell responses [5]. We find that immune senescence correlates with reduced CSR ability as a result of reduced transcription of Ig-isotypes and that this process starts around the age of fifty. This is in good agreement with reduced vaccine efficacy in the older population [4]–[6], [30]–[33]. Our results are also in concordance with recent findings that suggest a decrease in the number of mature activated B-cells and a decreasing ability for CSR in elderly populations above sixty years of age [34]. Mouse experiments suggest the molecular mechanism of ageing in B-cells to be driven by TNF-α and low-grade inflammation increasing with age. Subsequently this has a negative effect on transcription factor E47 and activation-induced cytidine deaminase and hence down-regulates CSR [35]. Noteworthy is the relatively early onset of immune senescence. This change in the immune system suggests itself to be influenced by hormonal changes at this period of life [36]. Our findings on immune senescence starting with the age of fifty calls for further investigation in this direction as well as the development of different treatment and vaccination procedures for the “Golden Ager”.

## Materials and Methods

### Medical history of healthy donors

The samples were collected from Caucasian donors living in the Berlin area by in.vent Diagnostica GmbH (Hennigsdorf, Germany) in the course of routine blood donation and represent leftovers from infectious disease screening. According to German Transfusion Law (Transfusionsgesetz - TFG) no ethical approval is necessary if material is collected during such a routine process, since no additional intervention is necessary. However, written informed consent of the donors is mandatory and has been obtained by all individuals for our specific research program. The identity of the donors was made anonymous by in.vent Diagnostica GmbH prior to sample transfer to the Max Planck Institute for Molecular Genetics. Prior blood donation, the donors stated to by free of any symptoms for at least 8 weeks and filled out a questionnaire with 37 questions concerning the following areas: previous vaccinations (<12 weeks), disease history in the areas of neurology (<10 years), otorhinolaryngology, lung, cardiovascular, liver, gastrointestinal tract (<10 years), pancreas, blood, cancer, kidney, endocrinology, rheumatism, gynecology (<10 years), eyes, serious infections (<10 years), skin, teeth, tropical diseases (e.g. malaria), pregnancy and allergy. Further, medication status was recorded for antibiotics, heart, blood clotting, diuretics, abstergent agents, glucocorticosteroids, anti-diabetic, thyroid, contraceptive, recreational and other drugs. Additionally, surgical interventions, alcohol consumption and smoking habits were recorded, as well as familial history of severe diseases, such as cancer and autoimmune disorders (Table 1). After questionnaire evaluation, only donors were finally included into the cohort who could answer the majority of these questions with a no. Exclusion criteria were predominantly fixed to disease or medication history, which could influence the immune status of the donor.

**Table 1:** Donor details and obtained numbers of sequences for analysis.

Donor ID	LC (No. of seq.)	HC (No. of seq.)	age	gender	Height in cm	Weight in kg	BMI&	surgeries
I192158-77	26257	65770	62	m	180	90	27.8	none
I192158-80*	15566	63697	49	m	170	70	24.2	none
I192158-95*	10763	54175	21	f	164	53	19.7	none
I192158-105*	11620	16019	24	m	187	87	24.9	nasal septum, wisdom tooth
I200091-002	755	75863	57	f	175	80	26.1	caecum, biliary
I200091-004	0	62891	30	f	174	65	21.5	ankle
I200091-017	13960	95743	54	f	162	66	25.1	tonsils
I200091-021	19834	128861	20	m	184	83	24.5	none
I200091-023	91528	127206	19	m	187	87	24.9	small surgery at index finger
I200091-024	79339	94201	60	f	167	70	25.1	tonsils, caecum, cyst at ovary
I200091-028	1196	46102	52	m	180	80	24.7	hand, knee, nose, throat, caecum, hernia inguinalis
I200091-030	13105	96308	22	m	180	86	26.5	none
I200091-032	2828	74597	20	f	167	57	20.4	nasal polyposis
I200091-038§	8804	45088	28	f	167	70	25.1	none

& BMI: body mass index;

* smoker;

§ familial history of cardiac defect.

### Sample preparation and sequencing

Reverse transcription and amplification of the mRNA was performed using the MINT cDNA synthesis kit (Evrogen, Russia). Immunoglobulin amplification was carried out in two independent reactions for heavy and light chain, respectively. Ig-class specific primers were pooled in an equal molar range to a final concentration of 10 pmol/µl to allow chain and donor specific ePCRs. ePCR conditions were as follows: initial heating at 98°C for 45 sec and 15 cycles elongation (98°C for 10 sec, 65°C for 20 sec and 72°C for 22 sec) and finally 72°C for 4 min. The amplicons were purified according a modified protocol [21] with a DNA purification kit (Roboklon, Germany) and the ePCR was repeated (additional 15 cycles). DNA was purified by 1.2% agarose gel electrophoresis, enzymatically cleaved with SfiI and ligated with self-made Roche454 adaptors containing appropriate SfiI-sites (Knaust et al, submitted). Ligated DNA was repurified by agarose gel electrophoresis and Agencourt AMPure XP. For sequencing, the libraries were treated according to the manufacturer's recommendation: bead-coupled amplification using the “GS FLX Titanium LV emPCR Kit” followed by sequencing applying “GS FLX Titanium Sequencing Kit XLR70” and “GS FLX Titanium PicoTiterPlate Kit”.

### Pattern search for Ig-isotype assignment

Isotypes were determined using a simplistic pattern matching approach using isotype-specific signatures found in the constant region of the different Ig-class (Table 2). Sequences of rearranged antibodies were tested against these signatures using the “Fuzznuc” program from the EMBOSS [37] suite (Version 6.1.0) with default parameters. The output was further processed using shell scripts to retrieve sequence identifiers to be saved in our DBMS for further analysis. The pattern IgG2-01-02-03-04 recognizes both IgG2 and IgG4 while IgG4-01-04 is specific for IgG4 alone – the intersection of sequences found by both patterns is considered as IgG4 and the relative complement of IgG2-01-02-03-04 in IgG4-01-04 as IgG2.

**Table 2:** Signature sequences used for the assignment of Ig-classes.

Ig-isotype with allele information	DNA-sequence
IgA1-01	GCAGAGGCTCA
IgA2-01-02-03	GTCGAGGCTCA
IgD-01-02	AGCCTTGGTGG
IgE-01-02	GCTCTGTGTGG
IgG2-01-02-03-04	GCTGTGCTCTCGGA
IgG3-01-02	AGAGGTGCTCCTGGAGCA
IgG4-01-04	AGGGCGGCTGTGCTC
IgM-01-03	GCGGATGCACTC
IgKC-01-02-03-04-05	GCAGCCACAGTT
IgLC1-2	GGCGGGAACA
IgLC3-7	GGTGGGAACA

### V(D)J assignment using IMGT/High V-Quest

Sequences (size selected, >380 bp) of the rearranged antibodies obtained were submitted to the IMGT/HighV-QUEST high throughput analysis portal with “allow insertions/deletions” option enabled. Output was filtered using following three steps: (i) We only considered sequences which had a complete set of V(D)J genes with a identity score greater than 85% and were successfully assigned to an Ig-class by pattern matching against isotype-specific signatures. (ii) IMGT/HighV-QUEST generated output at the allele level. For our analyses however, we classified sequences by gene. To convert alleles to genes, we applied a regular expression filter on the IMGT identifiers (Protocol S1). This filter merged different V, D or J-alleles into the respective genes and treated duplicated and unduplicated D-genes as similar. Additionally, a simple heuristic was employed to integrate some genes which were hardly distinguishable from each other and hence were often ambiguously assigned. (iii) As a last quality control, all sequences were excluded, which were assigned to more than one V, D or J-gene, respectively.

## Supporting Information

Figure S1 **VDJ recombination pattern distributions of 14 donors incorporating heavy chain isotype information.**
(PDF)

Figure S2 **VDJ rearrangements 100-fold over the median frequency of all VDJ recombination patterns within the cohort.**
(PDF)

Figure S3 **Variability as a function of unique VDJ recombination patterns in each isotype in proportion to all isotypes within donors.**
(PDF)

Figure S4 **Clustering of donors according to coincident appearance of most frequent VDJ rearrangements in IgM with all CSR-dependent isotypes.**
(PDF)

Figure S5 **Clustering of donors according to coincident appearance of most frequent VDJ rearrangements in IgM and IgG subisotypes.**
(PDF)

Figure S6 **Clustering of donors according to coincident appearance of most frequent VDJ rearrangements in IgG with subisotypes of IgG and with IgM.**
(PDF)

Figure S7 **Clustering of donors according to coincident appearance of most frequent VDJ rearrangements in IgM with IgA1 and IgA2.**
(PDF)

Protocol S1 **Regular expression filter used to integrate IMGT/High V-Quest alleles into genes.**
(PDF)

Text S1 **Supporting experimental data.**
(PDF)

Table S1 **Statistical analysis of relative amount of obtained sequences per isotype over the total number of sequences from all 14 donors.**
(PDF)

Table S2 **Statistical analysis of relative amount of obtained sequences per isotype over the total number of sequences from the young adult group.**
(PDF)

Table S3 **Statistical analysis of relative amount of obtained sequences per isotype over the total number of sequences from the elderly group.**
(PDF)

Table S4 **Unique VDJ recombination per isotype in proportion to all isotypes in all donors.**
(PDF)

Table S5 **Unique VDJ recombination per isotype in proportion to all isotypes in young donors.**
(PDF)

Table S6 **Unique VDJ recombination per isotype in proportion to all isotypes in elderly donors.**
(PDF)

Table S7 **Analysis of changes in the VDJ rearrangement pattern distribution by entropy over all donors.**
(PDF)

Table S8 **Analysis of changes in the VDJ rearrangement pattern distribution by entropy in the young adults.**
(PDF)

Table S9 **Analysis of changes in the VDJ rearrangement pattern distribution by entropy in the elderly.**
(PDF)

## References

[1] Schatz DG, Ji Y (2011) Recombination centres and the orchestration of V(D)J recombination. Nat Rev Immunol 11: 251–263. 21394103 10.1038/nri2941

[2] Stavnezer J, Guikema JE, Schrader CE (2008) Mechanism and regulation of class switch recombination. Annu Rev Immunol 26: 261–292. 18370922 10.1146/annurev.immunol.26.021607.090248 PMC2707252

[3] Schroeder HW Jr, Cavacini L (2010) Structure and function of immunoglobulins. J Allergy Clin Immunol 125: S41–52. 20176268 10.1016/j.jaci.2009.09.046 PMC3670108

[4] Frasca D, Blomberg BB (2011) Aging affects human B cell responses. J Clin Immunol 31: 430–435. 21318330 10.1007/s10875-010-9501-7 PMC5560853

[5] Aberle JH, Stiasny K, Kundi M, Heinz FX (2012) Mechanistic insights into the impairment of memory B cells and antibody production in the elderly. Age (Dordr) 10.1007/s11357-011-9371-9 PMC3592966 22282053

[6] Sasaki S, Sullivan M, Narvaez CF, Holmes TH, Furman D, et al (2011) Limited efficacy of inactivated influenza vaccine in elderly individuals is associated with decreased production of vaccine-specific antibodies. J Clin Invest 121: 3109–3119. 21785218 10.1172/JCI57834 PMC3148747

[7] Weinstein JA, Jiang N, White RA 3rd, Fisher DS, Quake SR (2009) High-throughput sequencing of the zebrafish antibody repertoire. Science 324: 807–810. 19423829 10.1126/science.1170020 PMC3086368

[8] Jiang N, Weinstein JA, Penland L, White RA 3rd, Fisher DS, et al (2011) Determinism and stochasticity during maturation of the zebrafish antibody repertoire. Proc Natl Acad Sci U S A 108: 5348–5353. 21393572 10.1073/pnas.1014277108 PMC3069157

[9] Wang Y, Jackson KJ, Gaeta B, Pomat W, Siba P, et al (2011) Genomic screening by 454 pyrosequencing identifies a new human IGHV gene and sixteen other new IGHV allelic variants. Immunogenetics 63: 259–265. 21249354 10.1007/s00251-010-0510-8

[10] Jackson KJ, Wang Y, Gaeta BA, Pomat W, Siba P, et al (2012) Divergent human populations show extensive shared IGK rearrangements in peripheral blood B cells. Immunogenetics 64: 3–14. 21789596 10.1007/s00251-011-0559-z

[11] Fischer N (2011) Sequencing antibody repertoires: the next generation. MAbs 3: 17–20. 21099370 10.4161/mabs.3.1.14169 PMC3038007

[12] Boyd SD, Gaeta BA, Jackson KJ, Fire AZ, Marshall EL, et al (2010) Individual variation in the germline Ig gene repertoire inferred from variable region gene rearrangements. J Immunol 184: 6986–6992. 20495067 10.4049/jimmunol.1000445 PMC4281569

[13] Glanville J, Zhai W, Berka J, Telman D, Huerta G, et al (2009) Precise determination of the diversity of a combinatorial antibody library gives insight into the human immunoglobulin repertoire. Proc Natl Acad Sci U S A 106: 20216–20221. 19875695 10.1073/pnas.0909775106 PMC2787155

[14] Prabakaran P, Chen W, Singarayan MG, Stewart CC, Streaker E, et al (2012) Expressed antibody repertoires in human cord blood cells: 454 sequencing and IMGT/HighV-QUEST analysis of germline gene usage, junctional diversity, and somatic mutations. Immunogenetics 64: 337–350. 22200891 10.1007/s00251-011-0595-8 PMC6953429

[15] Glanville J, Kuo TC, von Budingen HC, Guey L, Berka J, et al (2011) Naive antibody gene-segment frequencies are heritable and unaltered by chronic lymphocyte ablation. Proc Natl Acad Sci U S A 108: 20066–20071. 22123975 10.1073/pnas.1107498108 PMC3250199

[16] Wu YC, Kipling D, Leong HS, Martin V, Ademokun AA, et al (2010) High-throughput immunoglobulin repertoire analysis distinguishes between human IgM memory and switched memory B-cell populations. Blood 116: 1070–1078. 20457872 10.1182/blood-2010-03-275859 PMC2938129

[17] Boyd SD, Marshall EL, Merker JD, Maniar JM, Zhang LN, et al (2009) Measurement and clinical monitoring of human lymphocyte clonality by massively parallel VDJ pyrosequencing. Sci Transl Med 1: 12ra23. 10.1126/scitranslmed.3000540 PMC2819115 20161664

[18] Briney BS, Willis JR, Crowe JE Jr (2012) Human Peripheral Blood Antibodies with Long HCDR3s Are Established Primarily at Original Recombination Using a Limited Subset of Germline Genes. PLoS One 7: e36750. 22590602 10.1371/journal.pone.0036750 PMC3348910

[19] Zhu Z, Dimitrov DS (2009) Construction of a large naive human phage-displayed Fab library through one-step cloning. Methods Mol Biol 525: 129–142, xv. 19252833 10.1007/978-1-59745-554-1_6 PMC3423197

[20] Lim TS, Mollova S, Rubelt F, Sievert V, Dübel S, et al (2010) V-gene amplification revisited - An optimised procedure for amplification of rearranged human antibody genes of different isotypes. N Biotechnol 27: 108–117. 20083243 10.1016/j.nbt.2010.01.001

[21] Schütze T, Rubelt F, Repkow J, Greiner N, Erdmann VA, et al (2011) A streamlined protocol for emulsion polymerase chain reaction and subsequent purification. Anal Biochem 410: 155–157. 21111698 10.1016/j.ab.2010.11.029

[22] Margulies M, Egholm M, Altman WE, Attiya S, Bader JS, et al (2005) Genome sequencing in microfabricated high-density picolitre reactors. Nature 437: 376–380. 16056220 10.1038/nature03959 PMC1464427

[23] Lefranc MP, Giudicelli V, Ginestoux C, Jabado-Michaloud J, Folch G, et al (2009) IMGT, the international ImMunoGeneTics information system. Nucleic Acids Res 37: D1006–1012. 18978023 10.1093/nar/gkn838 PMC2686541

[24] Brochet X, Lefranc MP, Giudicelli V (2008) IMGT/V-QUEST: the highly customized and integrated system for IG and TR standardized V-J and V-D-J sequence analysis. Nucleic Acids Res 36: W503–508. 18503082 10.1093/nar/gkn316 PMC2447746

[25] Alamyar E, Giudicelli V, Li S, Duroux P, Lefranc MP (2012) IMGT/HighV-QUEST: the IMGT(R) web portal for immunoglobulin (IG) or antibody and T cell receptor (TR) analysis from NGS high throughput and deep sequencing. Immunome Res 8: 26.

[26] Arnaout RA (2005) Specificity and overlap in gene segment-defined antibody repertoires. BMC Genomics 6: 148. 16255770 10.1186/1471-2164-6-148 PMC1277825

[27] Maul RW, Saribasak H, Martomo SA, McClure RL, Yang W, et al (2011) Uracil residues dependent on the deaminase AID in immunoglobulin gene variable and switch regions. Nat Immunol 12: 70–76. 21151102 10.1038/ni.1970 PMC3653439

[28] Chao A, Shen T-J (2003) Nonparametric estimation of Shannon's index of diversity when there are unseen species in sample. Environmental and Ecological Statistics 10: 429–443.

[29] Burlington DB, Clements ML, Meiklejohn G, Phelan M, Murphy BR (1983) Hemagglutinin-specific antibody responses in immunoglobulin G, A, and M isotypes as measured by enzyme-linked immunosorbent assay after primary or secondary infection of humans with influenza A virus. Infect Immun 41: 540–545. 6874068 10.1128/iai.41.2.540-545.1983 PMC264675

[30] Gibson KL, Wu YC, Barnett Y, Duggan O, Vaughan R, et al (2009) B-cell diversity decreases in old age and is correlated with poor health status. Aging Cell 8: 18–25. 18986373 10.1111/j.1474-9726.2008.00443.x PMC2667647

[31] Weinberger B, Herndler-Brandstetter D, Schwanninger A, Weiskopf D, Grubeck-Loebenstein B (2008) Biology of immune responses to vaccines in elderly persons. Clin Infect Dis 46: 1078–1084. 18444828 10.1086/529197

[32] Stiasny K, Aberle JH, Keller M, Grubeck-Loebenstein B, Heinz FX (2012) Age affects quantity but not quality of antibody responses after vaccination with an inactivated flavivirus vaccine against tick-borne encephalitis. PLoS One 7: e34145. 22461903 10.1371/journal.pone.0034145 PMC3312914

[33] Weinberger B, Keller M, Fischer KH, Stiasny K, Neuner C, et al (2010) Decreased antibody titers and booster responses in tick-borne encephalitis vaccinees aged 50–90 years. Vaccine 28: 3511–3515. 20332047 10.1016/j.vaccine.2010.03.024

[34] Frasca D, Diaz A, Romero M, Landin AM, Blomberg BB (2011) Age effects on B cells and humoral immunity in humans. Ageing Res Rev 10: 330–335. 20728581 10.1016/j.arr.2010.08.004 PMC3040253

[35] Frasca D, Romero M, Diaz A, Alter-Wolf S, Ratliff M, et al (2012) A Molecular Mechanism for TNF-alpha-Mediated Downregulation of B Cell Responses. J Immunol 188: 279–286. 22116831 10.4049/jimmunol.1003964 PMC3700394

[36] Gameiro CM, Romao F, Castelo-Branco C (2010) Menopause and aging: changes in the immune system–a review. Maturitas 67: 316–320. 20813470 10.1016/j.maturitas.2010.08.003

[37] Rice P, Longden I, Bleasby A (2000) EMBOSS: the European Molecular Biology Open Software Suite. Trends Genet 16: 276–277. 10827456 10.1016/s0168-9525(00)02024-2

