# Supplementary material for: Onset of Immune Senescence Defined by Unbiased Pyrosequencing of Human Immunoglobulin mRNA Repertoires
Source: PLoS One. 2012 Nov 30;7(11):e49774. doi: 10.1371/journal.pone.0049774 (PMC3511497; doi:10.1371/journal.pone.0049774)
Supplement: Figure S1 — VDJ recombination pattern distributions of 14 donors incorporating heavy chain isotype information. (PDF) [file pone.0049774.s001.pdf]

Figure S1. VDJ recombination pattern distributions of 14 donors incorporating heavy chain isotype information.

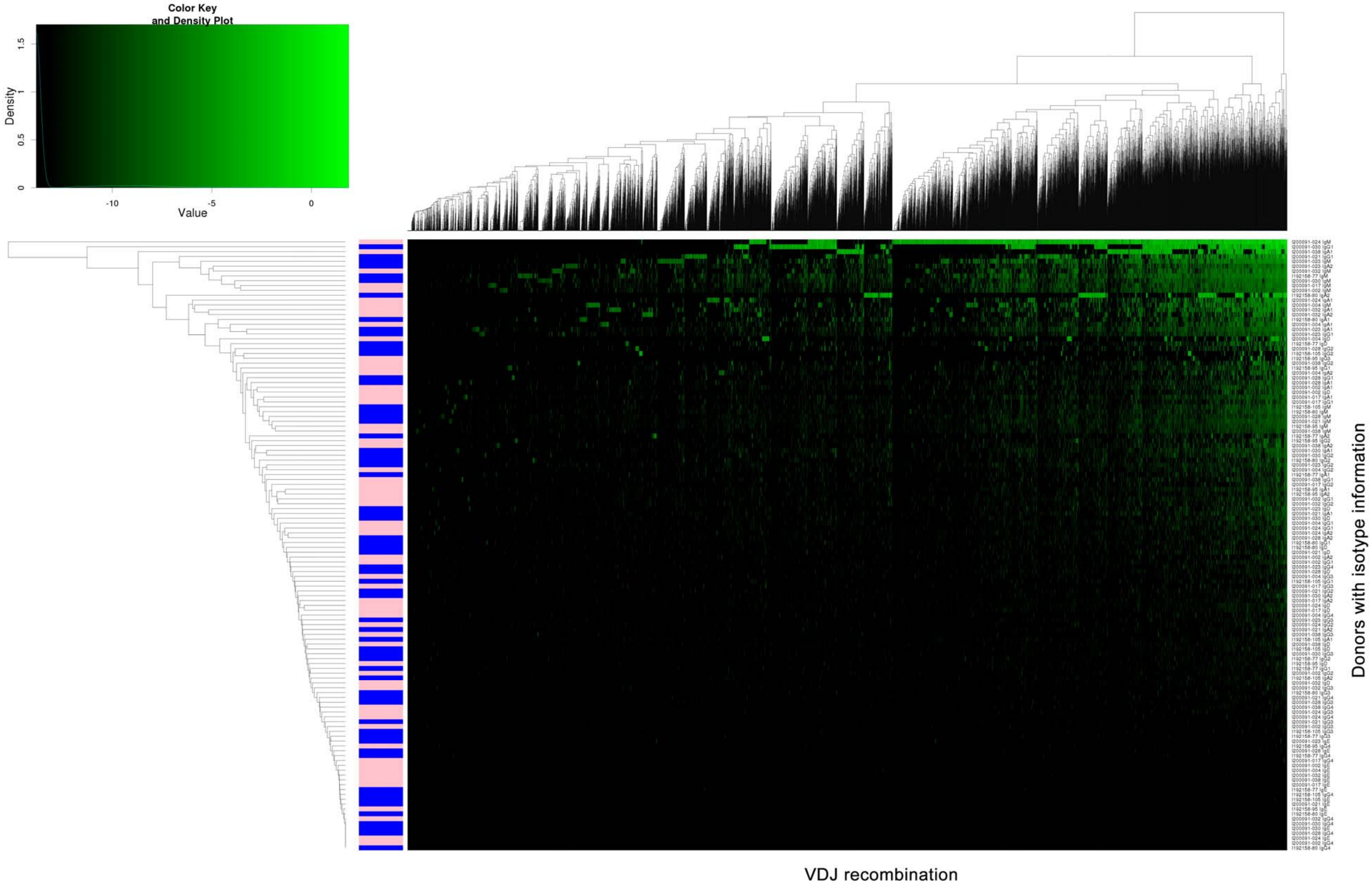

Heatmap shows relative frequency of VDJs recombination patterns in columns versus donors including antibody isotype information in rows. Gender of the donors is represented by blue and pink colors for male and female, respectively. Individual VDJ counts were normalized by total number of sequences for each donor. The distribution of VDJ frequencies showed an exponential distribution. Hence, coloring was applied to a log-transformation of the normalized frequencies *F* as ln(*F* + 1e-6) and visualized with increasing intensity from black to lime. Row and column dendograms use euclidean distance.
