# Supplementary material for: Onset of Immune Senescence Defined by Unbiased Pyrosequencing of Human Immunoglobulin mRNA Repertoires
Source: PLoS One. 2012 Nov 30;7(11):e49774. doi: 10.1371/journal.pone.0049774 (PMC3511497; doi:10.1371/journal.pone.0049774)
Supplement: Figure S2 — VDJ rearrangements 100-fold over the median frequency of all VDJ recombination patterns within the cohort. (PDF) [file pone.0049774.s002.pdf]

Figure S2. VDJ rearrangements 100-fold over the median frequency of all VDJ recombination patterns within the cohort.

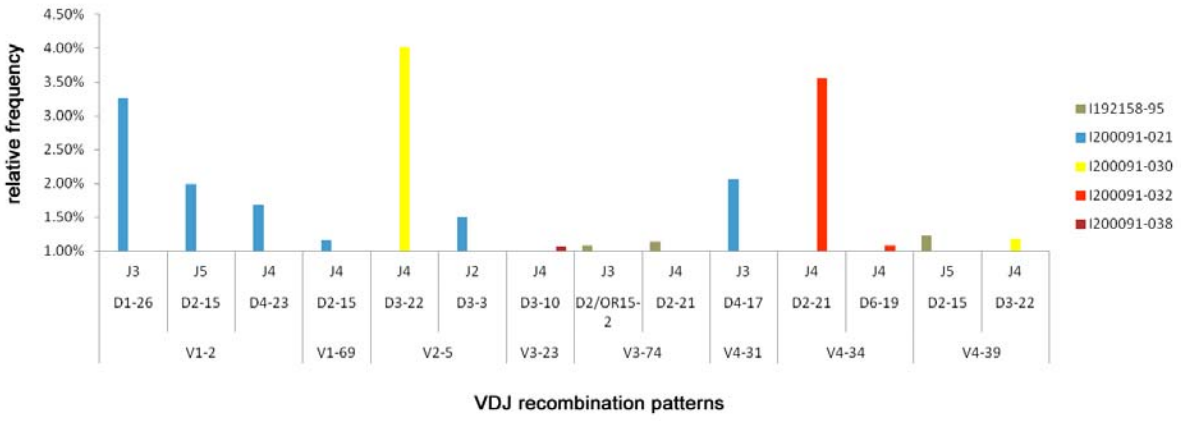

Graph represents all individual VDJ rearrangements with a frequency >1.07 % of all donors.
