# Supplementary material for: Onset of Immune Senescence Defined by Unbiased Pyrosequencing of Human Immunoglobulin mRNA Repertoires
Source: PLoS One. 2012 Nov 30;7(11):e49774. doi: 10.1371/journal.pone.0049774 (PMC3511497; doi:10.1371/journal.pone.0049774)
Supplement: Figure S3 — Variability as a function of unique VDJ recombination patterns in each isotype in proportion to all isotypes within donors. (PDF) [file pone.0049774.s003.pdf]

Figure S3. Variability as a function of unique VDJ recombination patterns in each isotype in proportion to all isotypes within donors.

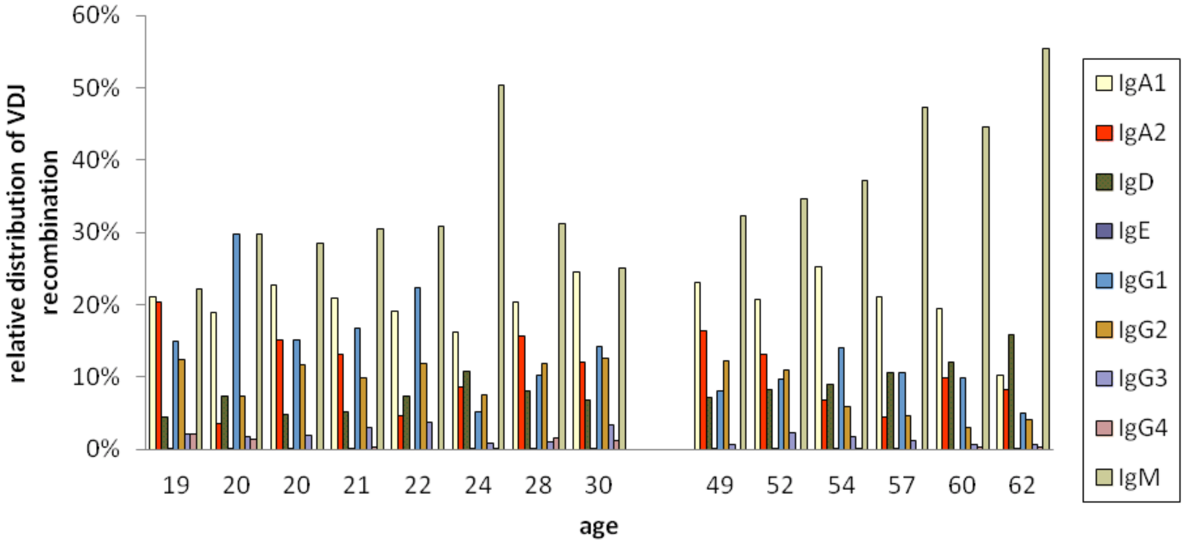

Variability was defined as the percentage of VDJs covered by a distinct antibody type in each donor. The variability V_{AD} for antibody type A and donor D was calculated from the number of occurring VDJs n_{AD} and the total number of occurring VDJs in the donor D n_D as n_{AD}/n_D.
