# Supplementary material for: Onset of Immune Senescence Defined by Unbiased Pyrosequencing of Human Immunoglobulin mRNA Repertoires
Source: PLoS One. 2012 Nov 30;7(11):e49774. doi: 10.1371/journal.pone.0049774 (PMC3511497; doi:10.1371/journal.pone.0049774)
Supplement: Figure S4 — Clustering of donors according to coincident appearance of most frequent VDJ rearrangements in IgM with all CSR-dependent isotypes. (PDF) [file pone.0049774.s004.pdf]

**Figure S4. Clustering of donors according to coincident appearance of most frequent VDJ rearrangements in IgM with all CSR-dependent isotypes.**

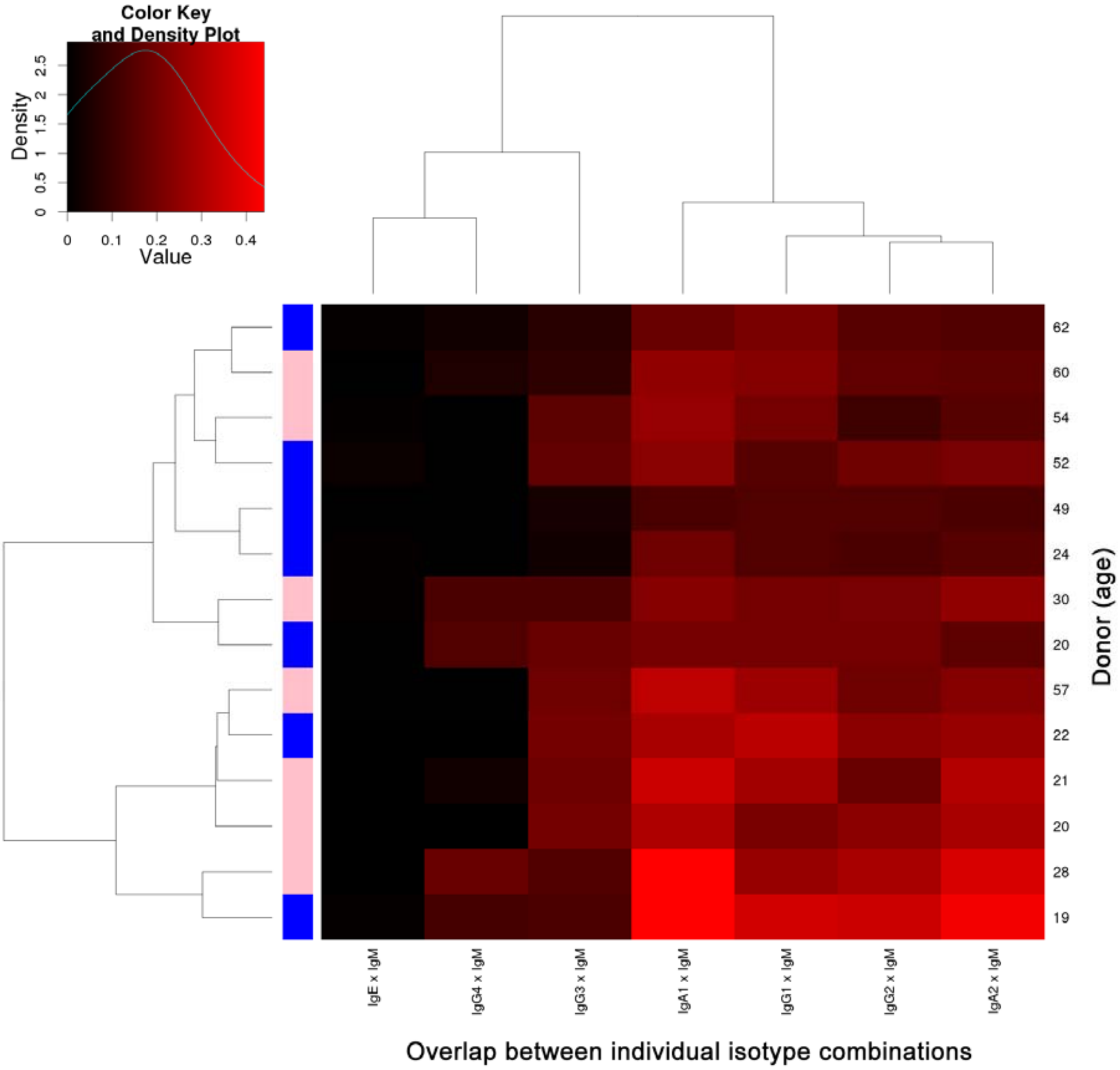


First, the hundred most frequently occurring VDJ recombination for each donor and antibody isotype (or less if there were less than hundred different VDJs) was determined. For each donor the overlap between each pair of antibodies was quantified using the formula $n_{both}/\max(n_A, n_B)$, where $n_{both}$ is the number of VDJs present in both sets of most frequently occurring VDJs and $n_A$ and $n_B$ are the sizes of the two sets. Gender of the donors is represented by blue and pink colors for male and female, respectively. The age of the donor is recorded on the right. Row and column dendograms use euclidean distance.
