# Supplementary material for: Onset of Immune Senescence Defined by Unbiased Pyrosequencing of Human Immunoglobulin mRNA Repertoires
Source: PLoS One. 2012 Nov 30;7(11):e49774. doi: 10.1371/journal.pone.0049774 (PMC3511497; doi:10.1371/journal.pone.0049774)
Supplement: Figure S5 — Clustering of donors according to coincident appearance of most frequent VDJ rearrangements in IgM and IgG subisotypes. (PDF) [file pone.0049774.s005.pdf]

Figure S5. Clustering of donors according to coincident appearance of most frequent VDJ rearrangements in IgM and IgG subisotypes.

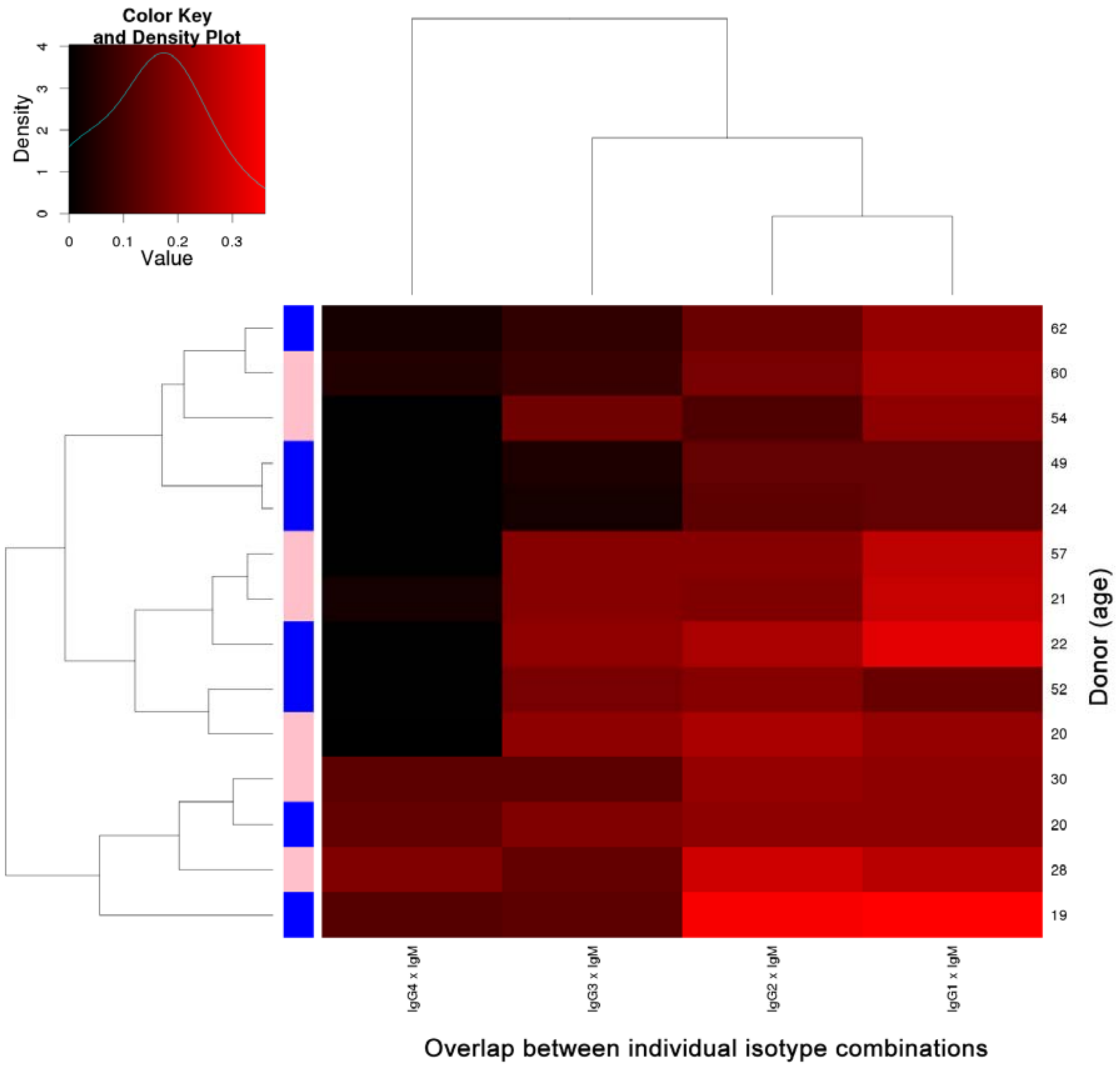

The heatmap was generated as described before, with considering only the shown overlap pairs. Gender of the donors is represented by blue and pink colors for male and female, respectively. The age of the donor is recorded on the right. Row and column dendograms use euclidean distance.
