# Supplementary material for: Onset of Immune Senescence Defined by Unbiased Pyrosequencing of Human Immunoglobulin mRNA Repertoires
Source: PLoS One. 2012 Nov 30;7(11):e49774. doi: 10.1371/journal.pone.0049774 (PMC3511497; doi:10.1371/journal.pone.0049774)
Supplement: Figure S6 — Clustering of donors according to coincident appearance of most frequent VDJ rearrangements in IgG with subisotypes of IgG and with IgM. (PDF) [file pone.0049774.s006.pdf]

Figure S6. Clustering of donors according to coincident appearance of most frequent VDJ rearrangements in IgG with subisotypes of IgG and with IgM.

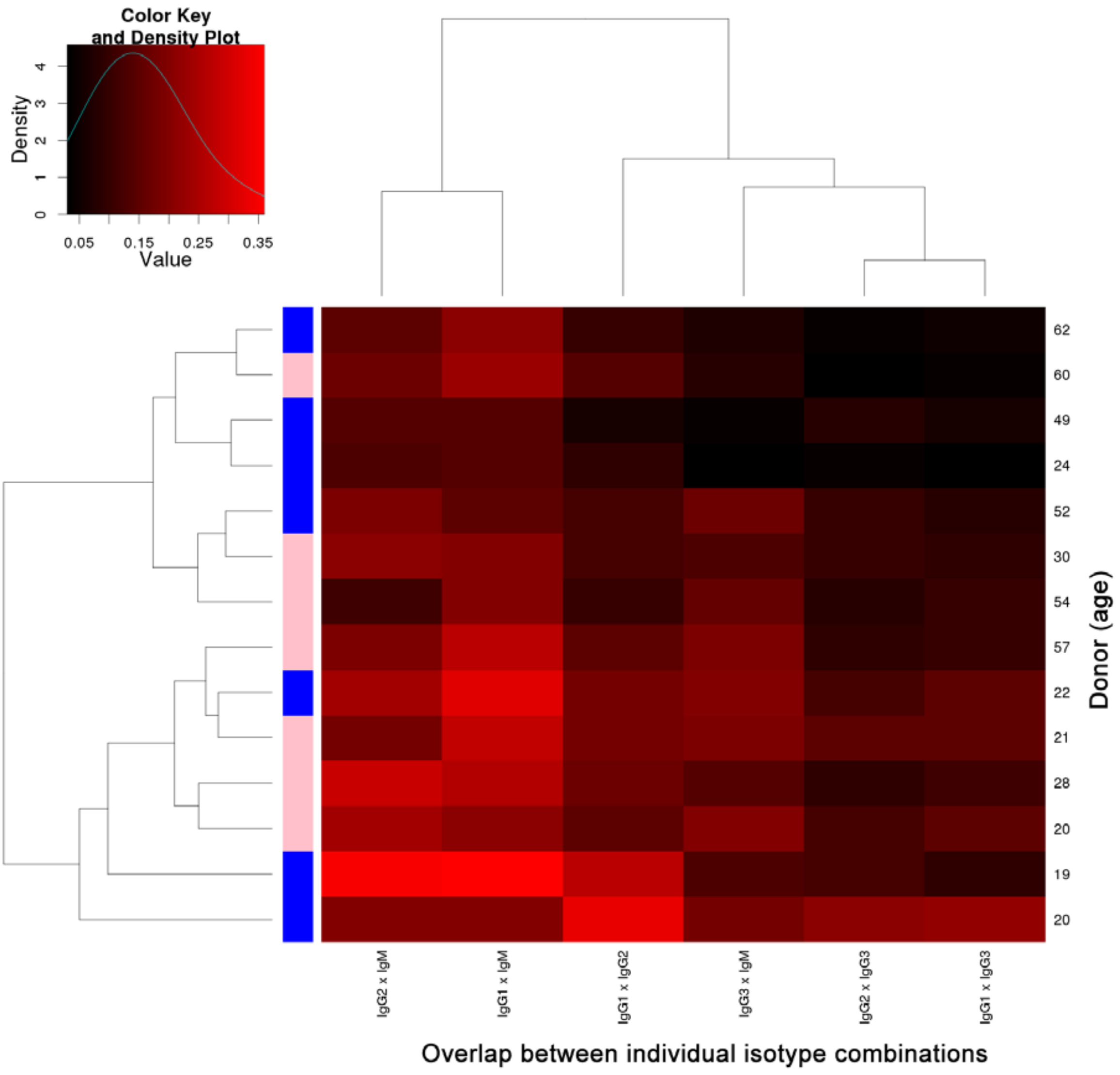

The heatmap was generated as described before, considering only the shown overlap pairs. Gender of the donors is represented by blue and pink colors for male and female, respectively. The age of the donor is recorded on the right. Row and column dendograms use euclidean distance.
