# Supplementary material for: Onset of Immune Senescence Defined by Unbiased Pyrosequencing of Human Immunoglobulin mRNA Repertoires
Source: PLoS One. 2012 Nov 30;7(11):e49774. doi: 10.1371/journal.pone.0049774 (PMC3511497; doi:10.1371/journal.pone.0049774)
Supplement: Protocol S1 — Regular expression filter used to integrate IMGT/High V-Quest alleles into genes. (PDF) [file pone.0049774.s008.pdf]

Supplementary Protocol S1: Regular expression filter used to integrate IMGT/High V-Quest alleles into genes. Filtering was performed at the database level using the following PL/SQL-function:

```
DECLARE
bfr varchar;
BEGIN
-- this regular expression matches IMGT identifiers without the allele part
bfr := substring(seq,'(IG[KHL][VDJ][0-9]??<1,2??>|IG[KHL][VDJ][0-
9]??<1,2??>D??<0,1??>-[hdcfba0-9]??<1,3??>|IG[KHL][VDJ][0-9]??<1,2??>-[0-
9]??<1,3??>-[0-9]??<0,2??>|IG[KHL][VDJ][0-9]??<1,2??>-NL[0-
9]??<1,3??>|IG[KHL][VDJ][0-9]??<1,2??>/OR[Y0-9]??<0,2??>-[0-9]??<1,3??>)');
-- SPECIAL CASES
-- Some genes turned out to be close variants of others.
-- Such variants were treated as one gene and were merged
-- into their most common "relative" because they often lead to ambigous
-- assignments in IMGT highVQuest runs
IF bfr = 'IGHV3-30-3' THEN
  bfr := 'IGHV3-30';
END IF;
IF bfr = 'IGHV3-NL1' THEN
 bfr := 'IGHV3-30';
END IF;
-- These IGHV4-30 subvariants are hardly distinguishable
-- hence we treat them as generic IGHV4-30
IF bfr = 'IGHV4-30-2' OR bfr = 'IGHV4-30-4' THEN
 bfr := 'IGHV4-30';
END IF;
-- IGHV4/OR15-8 is a variant from papua neuguinea and
-- matches to IGHV4-4 in an imgt search, hence we treat it as IGHV4-4
IF bfr = 'IGHV4/OR15-8' THEN
  bfr := 'IGHV4-4';
END IF;
-- IGHV3/OR16-6 is a variant from papua neuguinea and
-- matches to IGHV3-15 in an imgt search, hence we treat it as IGHV3-15
IF bfr = 'IGHV3/OR16-6' THEN
  bfr := 'IGHV3-15';
END IF;
-- checking for IGKV D variants
-- As we check for presence/absence we do not care for D
-- variants for now.
IF bfr ~ 'IGKV[0-9]*?D.[0-9]+' THEN
   bfr := replace(bfr,'D','');
END IF;
RETURN bfr;
END
```
