# Supplementary material for: Onset of Immune Senescence Defined by Unbiased Pyrosequencing of Human Immunoglobulin mRNA Repertoires
Source: PLoS One. 2012 Nov 30;7(11):e49774. doi: 10.1371/journal.pone.0049774 (PMC3511497; doi:10.1371/journal.pone.0049774)
Supplement: Text S1 — Supporting experimental data. (PDF) [file pone.0049774.s009.pdf]

Supplementary Text S1

Analysis of VDJ recombination patterns with isotype information.

Parallel to the general clustering according to heavy chain VDJ recombination patterns described in the main text, the 14 donors were also clustered with the observed VDJ recombination patterns being grouped according isotypes (Figure S1). This clustering revealed neither grouping according to age nor gender even after incorporation of isotype information. We found 6,685 unique VDJ recombination patterns (visualized in Figure 3A) of which most occurred only rarely in the different isotypes. The frequency of each was analyzed and the median frequency was calculated (0.017%). Some VDJ recombinations occur more frequently within donors (Figure S2).

Next, the relative number of unique VDJ recombination patterns per isotype in proportion to all isotypes for each donor were calculated (Figure S3). No age dependency in the young adults was seen and the relative number of unique VDJ recombinations for each isotype is comparable within this group. In the elderly however, age dependency was clearly observed with a gradual increase in relative numbers of VDJ recombination in IgM and IgD and a significant decrease in IgG2. The other isotypes in the elderly showed no distinct age-related decrease (see also Tables S4-S6). However, a strong correlation became evident by grouping the antibody isotypes in IgM/D and CSR isotypes as shown in Figure 4B.

Analysis of VDJ recombination patterns with CSR information.

We compared the VDJ recombination patterns within donors under the aspect of CSR by analyzing the overlap between VDJ recombinations in the different isotypes. For this, the results of the top 100 VDJ recombination within each isotype (IgM to IgAs/IgE/IgGs) were applied to cluster the donors by similarity (Figure S4). Using this approach revealed no gender specific clustering, but a tendency for clustering in young and elderly. Then, we compared the VDJ recombination patterns within the donors of the predominant class switch from IgM to IgG, again analyzing the top 100 VDJ recombination within each isotype by clustering the donors according to similarity (Figure S5). We detected no clustering relation according to gender and this time age-dependent clustering is not evident compared to Figure S4. Also, we analyzed the overlap between the most frequent 100 VDJ recombination patterns within the donors between all IgG sub-isotypes among themselves and IgM (Figure S6).

Again, no clustering relation according to gender was detected, but a similar tendency for clustering in young and elderly was observed as in Figure S4. Finally, we compared the VDJ recombination patterns within the donors between IgM and IgA1 and IgA2 analysing the top 100 VDJ recombination within each isotype (Figure S7). Clustering the donors by similarity revealed no relation according to age or gender.

Comparison of obtained sequences per isotype and their statistical analyses.

With our novel unbiased amplification and sequencing method for the first time a quantitative overview over all isotypes and their relative abundance is possible. We calculated for each donor the relative amount of obtained sequences per isotype over the total number of sequences per donor. Additionally we separated an immune reaction into initial response (IgM/D) and specific response (IgA/E/G) after class switch recombination (CSR). To assess possible age dependency, we calculated the relative abundance of all isotypes separately (Figure 4A) and as groups and analysed the relevance of our data statistically (Table S1). Over the total age period only IgM increase is significant and therefore also the combination IgM/D. Next, we assessed the age dependency separately in the young and the older adult group (Table S2 and S3, respectively), based on the relative abundance as above. In the young cohort no age dependency was observed and for all isotypes or groups no significant correlation or p-values were obtained. In the older adult group, isotypes IgD, IgM and IgG4 increase with increase of age, while the other decrease. However, only the increase of IgM and IgG4 is significant. Note that the data for IgG4 should be regarded with caution, as the overall number of sequences for this isotype are low in the analyzed set. In summary, the relative frequency of IgM sequences in the set is correlated with the increasing age of the donors in the set of elderly.

Changes in the distribution of unique recombination per isotype and their statistical analyses.

The variability as a function of unique VDJ recombinations per isotype in proportion to all isotypes for each donor was calculated (Figure S3). To assess age dependency, the changes of VDJ recombination proportion for each isotype in connection to the age of all donors were calculated as correlation and p-value (Table S4). A significant increase in IgD and IgM and a

decrease in IgG2 was observed in the cohort, however, the overall correlation to age is weak. When the analysis was repeated only on the data obtained from the young cohort, no age dependency was observed (Table S5). Next, we analyzed the elderly group on its own (Table S6). This revealed a significant increase and a strong correlation for IgD and IgM and a significant decrease in IgG2 to correlate with increasing age of the donors. The other isotypes showed mainly a slight, not significant reduction. When analyzing the two groups (initial response (IgM/D) and specific response (IgA/E/G)) a strong correlation between increase in IgM/D and decrease in IgA/E/G is seen with age.
