# Supplementary material for: Onset of Immune Senescence Defined by Unbiased Pyrosequencing of Human Immunoglobulin mRNA Repertoires
Source: PLoS One. 2012 Nov 30;7(11):e49774. doi: 10.1371/journal.pone.0049774 (PMC3511497; doi:10.1371/journal.pone.0049774)
Supplement: Table S1 — Statistical analysis of relative amount of obtained sequences per isotype over the total number of sequences from all 14 donors. (PDF) [file pone.0049774.s010.pdf]

Table S1. Statistical analysis of relative amount of obtained sequences per isotype over the total number of sequences from all 14 donors.

isotypes	correlation	p-value
IgA1	-0.20284	0.48675
IgA2	-0.32861	0.25132
IgD	0.58232	0.02889
IgE	0.24094	0.40666
IgG1	-0.45860	0.09908
IgG2	-0.47966	0.08262
IgG3	-0.43607	0.11906
IgG4	-0.44497	0.11086
IgM	0.70830	0.00458
IgM + IgD	0.70644	0.00474
IgA + IgE + IgG	-0.70644	0.00474

For all nine isotypes and groups correlation and p-values according a linear model fit (F-test) were calculated for age dependency.
