# Supplementary material for: Onset of Immune Senescence Defined by Unbiased Pyrosequencing of Human Immunoglobulin mRNA Repertoires
Source: PLoS One. 2012 Nov 30;7(11):e49774. doi: 10.1371/journal.pone.0049774 (PMC3511497; doi:10.1371/journal.pone.0049774)
Supplement: Table S2 — Statistical analysis of relative amount of obtained sequences per isotype over the total number of sequences from the young adult group. (PDF) [file pone.0049774.s011.pdf]

Table S2. Statistical analysis of relative amount of obtained sequences per isotype over the total number of sequences from the young adult group.

isotype	correlation	p-value
IgA1	0.34921	0.39651
IgA2	-0.11493	0.78640
IgD	0.52678	0.17980
IgE	0.12509	0.76790
IgG1	-0.37555	0.35926
IgG2	0.30270	0.46615
IgG3	0.31290	0.45049
IgG4	0.35856	0.38311
IgM	0.22724	0.58837
IgM + IgD	0.24762	0.55434
IgA + IgE + IgG	-0.24762	0.55434

For all nine isotypes and groups correlation and p-values according a linear model fit (F-test) were calculated for age dependency.
