# Supplementary material for: Onset of Immune Senescence Defined by Unbiased Pyrosequencing of Human Immunoglobulin mRNA Repertoires
Source: PLoS One. 2012 Nov 30;7(11):e49774. doi: 10.1371/journal.pone.0049774 (PMC3511497; doi:10.1371/journal.pone.0049774)
Supplement: Table S3 — Statistical analysis of relative amount of obtained sequences per isotype over the total number of sequences from the elderly group. (PDF) [file pone.0049774.s012.pdf]

Table S3. Statistical analysis of relative amount of obtained sequences per isotype over the total number of sequences from the elderly group.

isotype	correlation	p-value
IgA1	-0.56552	0.24215
IgA2	-0.51642	0.29423
IgD	0.46762	0.34970
IgE	-0.14161	0.78901
IgG1	-0.42357	0.40264
IgG2	-0.86254	0.02704
IgG3	-0.54596	0.26243
IgG4	0.84907	0.03245
IgM	0.85822	0.02873
IgM + IgD	0.85685	0.02927
IgA + IgE + IgG	-0.85685	0.02927

For all nine isotypes and groups correlation and p-values according a linear model fit (F-test) were calculated for age dependency.
