# Supplementary material for: Onset of Immune Senescence Defined by Unbiased Pyrosequencing of Human Immunoglobulin mRNA Repertoires
Source: PLoS One. 2012 Nov 30;7(11):e49774. doi: 10.1371/journal.pone.0049774 (PMC3511497; doi:10.1371/journal.pone.0049774)
Supplement: Table S4 — Unique VDJ recombination per isotype in proportion to all isotypes in all donors. (PDF) [file pone.0049774.s013.pdf]

Table S4. Unique VDJ recombination per isotype in proportion to all isotypes in all donors.

isotypes	correlation	p-value
IgA1	-0.15983	0.58521
IgA2	-0.23863	0.41131
IgD	0.73313	0.00285
IgE	0.23648	0.26855
IgG1	-0.56822	0.03401
IgG2	-0.64336	0.01305
IgG3	-0.54214	0.04521
IgG4	-0.47527	0.08928
IgM	0.64514	0.01272
IgM + IgD	0.67501	0.00808
IgA + IgE + IgG	-0.67501	0.00808

Correlations were calculated using the Pearson rank method and linear dependencies were evaluated by standard linear model fits. Significance of the intercept term was than quantified with an F-test.
