# Supplementary material for: Onset of Immune Senescence Defined by Unbiased Pyrosequencing of Human Immunoglobulin mRNA Repertoires
Source: PLoS One. 2012 Nov 30;7(11):e49774. doi: 10.1371/journal.pone.0049774 (PMC3511497; doi:10.1371/journal.pone.0049774)
Supplement: Table S5 — Unique VDJ recombination per isotype in proportion to all isotypes in young donors. (PDF) [file pone.0049774.s014.pdf]

Table S5. Unique VDJ recombination per isotype in proportion to all isotypes in young donors.

isotypes	correlation	p-value
IgA1	0.27138	0.51560
IgA2	0.01206	0.97739
IgD	0.44763	0.26607
IgE	0.57430	0.17752
IgG1	-0.45257	0.26018
IgG2	0.25725	0.53852
IgG3	-0.00097	0.99817
IgG4	-0.02829	0.95758
IgM	0.11277	0.79033
IgM + IgD	0.18371	0.66321
IgA + IgE + IgG	-0.18371	0.66321

Correlations were calculated using the Pearson rank method and linear dependencies were evaluated by standard linear model fits. Significance of the intercept term was than quantified with an F-test.
