# Supplementary material for: Onset of Immune Senescence Defined by Unbiased Pyrosequencing of Human Immunoglobulin mRNA Repertoires
Source: PLoS One. 2012 Nov 30;7(11):e49774. doi: 10.1371/journal.pone.0049774 (PMC3511497; doi:10.1371/journal.pone.0049774)
Supplement: Table S6 — Unique VDJ recombination per isotype in proportion to all isotypes in elderly donors. (PDF) [file pone.0049774.s015.pdf]

Table S6. Unique VDJ recombination per isotype in proportion to all isotypes in elderly donors.

isotypes	correlation	p-value
IgA1	-0.73945	0.09298
IgA2	-0.63579	0.17482
IgD	0.95275	0.00330
IgE	-0.08037	0.89777
IgG1	-0.32645	0.52771
IgG2	-0.91184	0.01132
IgG3	-0.41907	0.40820
IgG4	0.92849	0.24221
IgM	0.93712	0.00581
IgM + IgD	0.94895	0.00384
IgA + IgE + IgG	-0.94895	0.00384

Correlations were calculated using the Pearson rank method and linear dependencies were evaluated by standard linear model fits. Significance of the intercept term was than quantified with an F-test.
