# Supplementary material for: Onset of Immune Senescence Defined by Unbiased Pyrosequencing of Human Immunoglobulin mRNA Repertoires
Source: PLoS One. 2012 Nov 30;7(11):e49774. doi: 10.1371/journal.pone.0049774 (PMC3511497; doi:10.1371/journal.pone.0049774)
Supplement: Table S7 — Analysis of changes in the VDJ rearrangement pattern distribution by entropy over all donors. (PDF) [file pone.0049774.s016.pdf]

Table S7. Analysis of changes in the VDJ rearrangement pattern distribution by entropy over all donors.

isotypes	correlation	p-value
IgA1	0.01049	0.97160
IgA2	-0.26130	0.36686
IgD	0.31680	0.26977
IgE	0.18646	0.94504
IgG1	-0.44889	0.10738
IgG2	-0.66682	0.00920
IgG3	-0.54034	0.04607
IgG4	-0.36296	0.41348
IgM	0.33073	0.24810
