# Supplementary material for: Onset of Immune Senescence Defined by Unbiased Pyrosequencing of Human Immunoglobulin mRNA Repertoires
Source: PLoS One. 2012 Nov 30;7(11):e49774. doi: 10.1371/journal.pone.0049774 (PMC3511497; doi:10.1371/journal.pone.0049774)
Supplement: Table S8 — Analysis of changes in the VDJ rearrangement pattern distribution by entropy in the young adults. (PDF) [file pone.0049774.s017.pdf]

## Table S8. Analysis of changes in the VDJ rearrangement pattern distribution by entropy in the young adults.

| isotypes | correlation | p-value |
|---|---|---|
| IgA1 | 0.05655 | 0.89419 |
| IgA2 | 0.10641 | 0.80198 |
| IgD | -0.24102 | 0.56528 |
| IgE | 0.59089 | 0.40911 |
| IgG1 | -0.28189 | 0.49879 |
| IgG2 | -0.05481 | 0.89744 |
| IgG3 | -0.22140 | 0.59825 |
| IgG4 | -0.07953 | 0.88096 |
| IgM | -0.58855 | 0.12482 |
