# Supplementary material for: Onset of Immune Senescence Defined by Unbiased Pyrosequencing of Human Immunoglobulin mRNA Repertoires
Source: PLoS One. 2012 Nov 30;7(11):e49774. doi: 10.1371/journal.pone.0049774 (PMC3511497; doi:10.1371/journal.pone.0049774)
Supplement: Table S9 — Analysis of changes in the VDJ rearrangement pattern distribution by entropy in the elderly. (PDF) [file pone.0049774.s018.pdf]

Table S9. Analysis of changes in the VDJ rearrangement pattern distribution by entropy in the elderly.

isotypes	correlation	p-value
IgA1	-0.74272	0.09077
IgA2	-0.64811	0.16395
IgD	0.94074	0.00516
IgE	-0.67353	0.32647
IgG1	-0.31234	0.54673
IgG2	-0.92320	0.00862
IgG3	-0.40495	0.42578
IgG4	NA	NA
IgM	0.84451	0.03439
